# Surface Electromyography Reveals Subject-Specific Alterations in Lumbar Flexion–Relaxation Following Prolonged Cycling in Pain-Free Road Cyclists

**DOI:** 10.3390/s26072214

**Published:** 2026-04-03

**Authors:** David Arriagada-Tarifeño, Natalia Belmar, Maricel Cabezas, Javiera Ceballos, Nicole Cedeño, Iver Cristi-Sánchez, Nicolás Casanova, Sebastián Chávez, Britam Gómez

**Affiliations:** 1 Laboratory of Applied Neuromechanics, School of Kinesiology, Faculty of Medical Sciences, Universidad de Santiago de Chile, Chacabuco N°675, Santiago 8320000, Chile; david.arriagada@usach.cl (D.A.-T.); natalia.belmar@usach.cl (N.B.); maricel.cabezas@usach.cl (M.C.); javiera.ceballos@usach.cl (J.C.); nicole.cedeno@usach.cl (N.C.); iver.cristi@usach.cl (I.C.-S.); 2 Department of Biomedical Engineering, Faculty of Engineering, Universidad de Santiago de Chile, Av. Libertador Bernardo O’Higgins N°3363, Santiago 9170022, Chile; nicolas.casanova@usach.cl (N.C.); sebastian.chavez.o@usach.cl (S.C.)

**Keywords:** surface electromyography, flexion–relaxation phenomenon, road cycling, ventilatory thresholds, lumbar neuromuscular control, physiological sensing

## Abstract

Low back pain is common in road cyclists and has been associated with prolonged lumbar flexion during cycling. The flexion–relaxation (FR) phenomenon reflects neuromuscular control of the lumbar spine, but its response to prolonged cycling under physiologically individualized conditions remains unclear. Thirty-one pain-free road cyclists completed a laboratory protocol in which exercise intensity was prescribed at 50% of the range between the first and second ventilatory thresholds (VT1 and VT2). Surface electromyography (sEMG) was recorded during trunk flexion extension tasks performed before and after a 60 min cycling trial. FR responses were characterized at both the individual and group levels using the flexion–relaxation ratio (FRR), descriptive classification of altered patterns, and exploratory estimates of mean change, effect size, and 95% confidence intervals. Four cyclists (12.9%; 95% CI: 3.6–29.8%) exhibited altered FR responses: three showed persistent alterations already present before cycling, and one showed an exercise-associated alteration. Group-level changes were minimal (effect sizes: −0.20 to 0.04). These findings suggest that prolonged cycling under controlled physiological load primarily reveals heterogeneous subject-specific neuromuscular patterns rather than a uniform average response. FR assessment using sEMG may therefore be useful as a complementary tool for identifying individual neuromuscular behavior in pain-free cyclists.

## 1. Introduction

Road cycling is associated with substantial health benefits; however, the mechanical demands inherent to this sport may predispose athletes to musculoskeletal conditions, particularly low back pain (LBP) [1,2,3,4]. Epidemiological evidence indicates that between 30% and 60% of cyclists experience LBP at some point during their sporting career [5,6,7], positioning this condition as a leading cause of reduced performance and clinical consultation among cyclists [8]. From a biomechanical perspective, cycling is characterized by a sustained flexed lumbar posture combined with repetitive lower-limb movements [9,10], which increases the loading on lumbar spinal segments and influences lumbopelvic mobility [11]. Prolonged exposure to sustained lumbar flexion has been associated with modifications in neuromuscular modulation of the lumbar extensors and may contribute to changes in sensorimotor behavior [12,13].

Low back pain is highly prevalent among road cyclists and has been associated with prolonged lumbar flexion during cycling. This posture increases mechanical loading on lumbar structures and may contribute to neuromuscular alterations even in asymptomatic athletes. Surface electromyography (sEMG) provides a non-invasive sensing method to quantify neuromuscular control of the lumbar spine during functional tasks. In this context, the flexion–relaxation (FR) phenomenon has been widely used to assess lumbar muscle activity patterns during trunk flexion–extension movements and to identify potential alterations in neuromuscular control.

The flexion–relaxation (FR) response represents an objective marker of lumbar neuromuscular behavior and is characterized by a marked inhibition of electromyographic (sEMG) activity of the lumbar extensors upon reaching maximal trunk flexion [14,15,16]. Alterations in this response—defined by reduced or absent sEMG silence in the expected suppression of muscle activity during the flexion–relaxation phase—are reflected by residual EMG activation when a substantial reduction would normally occur [17,18]. Modifications of the FR response have also been observed following sustained flexion tasks in asymptomatic individuals [19,20], indicating that measurable changes in neuromuscular behavior can occur in the absence of pain.

Despite the biomechanical complexity of cycling, relatively few studies have specifically characterized lumbar neuromuscular responses in this population [21,22,23]. The existing research has predominantly focused on lower-limb mechanics, pedaling efficiency, and performance outcomes, while the sensorimotor function of the lumbar spine has received comparatively limited attention [24,25,26]. Recent investigations employing sEMG and biomechanical approaches have begun to describe cycling-related neuromuscular responses, reinforcing the relevance of objective lumbar function assessment. However, no studies to date have evaluated FR responses before and after a prolonged physiologically individualized cycling task in pain-free cyclists.

In addition, previous studies have typically employed non-individualized exercise intensities or shorter-duration protocols, which may not adequately represent the physiological and mechanical demands of real-world cycling conditions [21,22,23,27]. This limitation may reduce the sensitivity to detect subtle or early neuromuscular responses, particularly in asymptomatic individuals.

Therefore, the present study aimed to investigate whether prolonged cycling performed under physiologically individualized conditions (based on ventilatory thresholds) is associated with modifications in the flexion–relaxation (FR) response in pain-free road cyclists. To address this, both group-level changes and individual response patterns were analyzed to provide a comprehensive characterization of lumbar neuromuscular behavior under controlled physiological loading.

We hypothesized that prolonged cycling would not produce substantial average changes at the group level but could be associated with heterogeneous individual responses, including both stable and exercise-associated alterations in FR behavior.

## 2. Materials and Methods

### 2.1. Study Design

A descriptive observational study was conducted, consisting of two evaluation sessions separated by an interval of 4 to 7 days. The first session aimed to determine individual ventilatory thresholds, while the second comprised the assessment of lumbar sensorimotor function before and after a prolonged cycling trial under laboratory conditions. All participants underwent a structured clinical interview prior to enrollment to confirm the absence of low back pain during the previous six months and to rule out sensorimotor disturbances or functional limitations.

#### Sample Size

The sample size was estimated considering the expected variability of sEMG-derived measures and the detection of small-to-moderate effect sizes, which indicated that a substantially larger sample would be required to achieve high statistical power for group-level comparisons. However, considering the experimental design—characterized by individualized physiological testing, prolonged cycling protocols, and multimodal signal acquisition—the final sample size (*n* = 31) was determined to ensure methodological consistency and data quality, in line with previous experimental studies in human neuromechanics employing comparable approaches [21,22,23,28].

All analyses were conducted using data from participants who completed the full protocol, while group-level descriptive statistics were computed for the non-altered subgroup.

### 2.2. Participants

Thirty-one recreational male road cyclists (mean age: 28.6 ± 7.7 years; body mass: 73.1 ± 10.2 kg; height: 1.72 ± 0.07 m), with an average of 6.1 ± 3.9 years of cycling experience and a weekly training volume of 9.8 ± 4.3 h, voluntarily participated in the study. A total of 35 cyclists initially volunteered, but four did not complete the full protocol and were therefore excluded from the final analysis. Of the four excluded participants, three did not complete the second evaluation due to scheduling or time constraints, and one discontinued the cycling trial due to fatigue. Importantly, none reported lumbar pain or discomfort during the protocol.

The inclusion criteria were a minimum of two years of road cycling experience and ≥ 6 h/week of regular training. The exclusion criteria included traumatic injuries or surgeries in the past year, cardiorespiratory disease, and structural spinal abnormalities. All participants were pain-free prior to testing and denied low back pain during their regular training over the previous six months. All procedures were approved by the Ethics Committee of the Universidad de Santiago de Chile (approval No. 306/2022) and were conducted in accordance with institutional ethical standards and international guidelines for research involving human participants. All participants provided written informed consent prior to voluntary participation.

### 2.3. Experimental Protocol

#### Cycling Equipment

All evaluations were performed using each participant’s personal bicycle, maintaining their habitual configuration. For mounting on the Wahoo KICKR V5 magnetic resistance trainer (Wahoo Fitness, Atlanta, GA, USA), the rear wheel was removed, the compatible axle and cassette installed, and the adapter adjusted to wheel size. Each participant verified drivetrain functionality before testing.

Heart rate was recorded with a Polar H10 chest strap (Polar Electro Oy, Kempele, Finland). Ventilation was measured using the Chaski wearable respiratory device (IC Innovations SpA, Santiago, Chile). Pedaling power was obtained from the Zwift virtual cycling platform (Zwift Inc., Long Beach, CA, USA), displayed on a laptop computer and projected onto a 60-inch screen positioned approximately 2 m in front of the participant. sEMG was recorded using the Delsys Trigno Wireless EMG System (Delsys Inc., Boston, MA, USA), with a sampling frequency of 1926 Hz for sEMG and 148 Hz for integrated triaxial accelerometry.

Bilateral electrodes were placed over the longissimus lumborum and multifidus muscles following SENIAM recommendations [29]. The skin was shaved and cleaned with isopropyl alcohol before sensor placement. It is important to note that due to the anatomical proximity and overlapping fiber orientation of these muscles, signal cross-talk cannot be completely excluded. Although electrode placement followed the established anatomical guidelines to improve recording selectivity, no advanced spatial filtering or high-density EMG techniques were used to quantify or minimize cross-talk. Consequently, the recorded signals should be interpreted as representing regional lumbar extensor muscle activity rather than activation of individual muscles.

### 2.4. Day 1: Determination of Ventilatory Thresholds

An incremental ramp test was performed on the Wahoo KICKR V5 trainer using the participant’s personal bicycle. The protocol consisted of 3 min at 50 W (warm-up), followed by an increase to 100 W to initiate the test, then increments of 20 W every 1 min 20 s until volitional exhaustion. Participants were allowed to freely choose the cadence and gear ratio but were required to remain seated, hands on the handlebars, and refrain from speaking to avoid interference with ventilation measurements. The test ended when the participant could not maintain the target power or voluntarily stopped pedaling. The first (VT1) and second (VT2) ventilatory thresholds were determined indirectly from the ventilation curve, heart rate, and power output, as previously validated [30].

### 2.5. Day 2: Lumbar Sensorimotor Assessment and 60 Min Cycling Trial

#### 2.5.1. Pre-Test Flexion–Relaxation (FR)

Participants, barefoot and in cycling apparel, performed three FR cycles (4 s flexion–hold–return) following audiovisual cues on a Galaxy Tab A7 Lite tablet (Samsung, Daegu, Republic of Korea), with 10 s rests between repetitions. Visual supervision and verbal instructions were used to ensure that participants performed maximal trunk flexion to the best of their ability.

#### 2.5.2. Maximal Voluntary Contraction (MVC)

From a 45° trunk-flexion position on a treatment table, participants performed three maximal isometric lumbar extensor contractions for 4 s with 30–60 s rests. These recordings were used for sEMG normalization.

#### 2.5.3. 60 Min Cycling Trial

The bicycle was remounted on the Wahoo KICKR V5 trainer, and both the heart rate monitor and trainer were connected to Zwift via Bluetooth. Participants maintained the brake/shift hood grip throughout the test and were prohibited from standing or changing hand positions to ensure a sustained trunk-flexed posture. Hydration and sweat wiping were permitted with one hand for brief intervals. After a 5 min warm-up (80–100 W), participants completed 60 min of continuous cycling at a power equivalent to 50% of the range between VT1 and VT2. Every 10 min, readiness to continue was verbally confirmed. The test ended in cases of excessive fatigue or discomfort.

#### 2.5.4. Post-Test Flexion–Relaxation

Within 3 min of completing the trial, the FR test was repeated under identical conditions. Signal integrity was verified, and electrodes were re-installed if necessary. As the pre-test FR, this was supervised by a health area professional to ensure the right execution of the 90 degree back bending.

### 2.6. sEMG and Signal Processing

#### 2.6.1. Time Analysis

sEMG and accelerometry signals were processed following a standardized and previously validated protocol [28]. sEMG signals were sampled at 1926 Hz and filtered using a fourth-order Butterworth band-pass filter (20–450 Hz) applied in a zero-phase forward–backward manner to minimize phase distortion. Motion-related outliers were identified and corrected using a Hampel filter with a window length of 200 ms, a threshold of 3 median absolute deviations (MAD) and a scaling factor of 1.4826.

The sEMG envelope was computed using a moving root mean square (RMS) calculated over 250 ms windows:

(1) $$RMS\left(t\right)=\sqrt{\frac{1}{N}\sum_{i=1}^{N}x^{2}\left(t-i\right)},$$

where x(t) represents the filtered sEMG signal, and *N* corresponds to the number of samples within the analysis window.

Each sEMG channel was normalized to its respective maximal voluntary contraction (MVC):

(2) $$sEMG_{\mathrm{norm}}\left(t\right)=\frac{RMS(t)}{MVC}\times 100.$$

**Figure 1:**
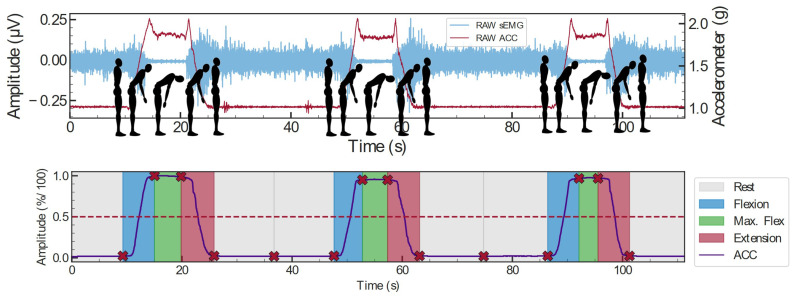
Flexion–relaxation (FR) test protocol and representative electromyographic response. Top figure illustrates the experimental sequence including rest, forward flexion, maximal flexion, and extension phases used for automatic segmentation of sEMG signals. Bottom figure illustrate the filtered acceleration with the transition events (“X” marks) [28].

Automatic segmentation of the flexion–relaxation (FR) test phases (rest, flexion, maximal flexion, and extension) was performed using the vertical accelerometer signal sampled at 148 Hz. Phase boundaries were detected using threshold-based criteria combined with local extrema adjustment to refine temporal transitions (Figure 1).

The FR response was quantified using the flexion–relaxation ratio (FRR), defined as

(3) $$FRR=\frac{{\bar{sEMG}}_{\mathrm{flexion}}}{{\bar{sEMG}}_{\max\;\mathrm{flexion}}},$$

where ${\bar{sEMG}}_{\mathrm{flexion}}$ and ${\bar{sEMG}}_{\max\;\mathrm{flexion}}$ represent the mean normalized sEMG activity during trunk flexion and maximal flexion phases, respectively. In this analysis, if the FRR values approach 1, this indicates that muscle activity during the relaxation phase is similar in magnitude to that during the active flexion phase, therefore suggesting minimal or absent flexion–relaxation behavior.

The absence of myoelectric silence was defined as sEMG activity during maximal flexion exceeding the predefined threshold, operationally defined as

(4) $${\bar{sEMG}}_{\max\;\mathrm{flexion}}>\theta ,$$

where θ corresponds to a predefined threshold (resting activity +2 standard deviations).

#### 2.6.2. Time–Frequency Analysis

sEMG signals are analyzed using Morlet Continuous Wavelet Transform (CWT) to dissect the signal’s time–frequency components (Equation (5)).

(5) $$W\left(s,\tau \right)=\int_{-\infty }^{\infty }x\left(t\right)\cdot \psi_{s,\tau }^{*}\left(t\right)\;dt$$

Here, W(s,τ) denotes wavelet coefficients at scale *s* and shift τ.

The power of the wavelet coefficients is calculated as shown in Equation (6):

(6) $$P\left(s,\tau \right)={\left|W\left(s,\tau \right)\right|}^{2}$$

Here, P(s,τ) is the power of the wavelet coefficients at scale *s* and time shift τ.

To better represent muscle activation events, a baseline power is determined using the first second of the measurement, representing a resting state, as shown in Equation (7):

(7) $$P_{\mathrm{baseline}}=\frac{1}{N}\sum_{i=1}^{N}P\left(s,\tau_{i}\right)$$

Here, $P_{\mathrm{baseline}}$ is the average power during the baseline period, *N* is the number of points in the baseline period, and $P(s,\tau_{i})$ is the power at each point *i* within the baseline period.

Then, the power spectrum is normalized relative to the baseline power, as shown in Equation (8).

(8) $$P_{\mathrm{norm}}\left(s,\tau \right)=\frac{P(s,\tau )}{P_{\mathrm{baseline}}}$$

where $P_{\mathrm{norm}}\left(s,\tau \right)$ is the normalized power relative to the baseline power.

**Figure 2:**
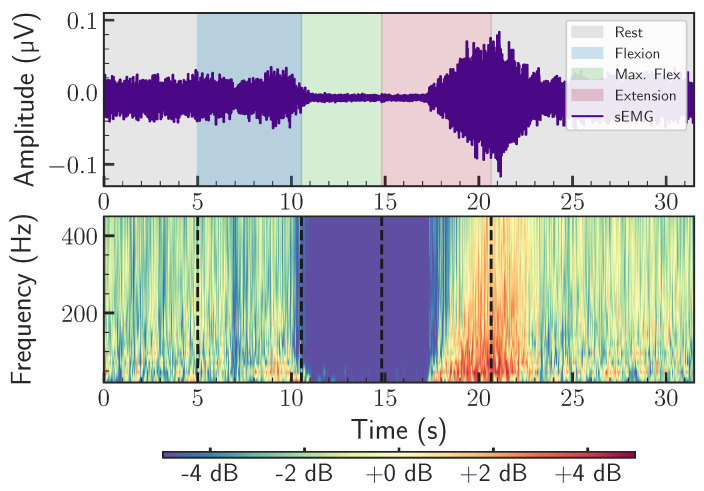
Time-frequency power plot in decibels. (**Top**) Averaged sEMG of the left longissimus. (**Bottom**) Time-frequency power plot of the wavelet transform [28].

Finally, the normalized power is expressed in decibels (dB) for improved visualization, as shown in Equation (9) (see the Time–Frequency Power plot (TFP) shown in Figure 2).

(9) $$P_{\mathrm{dB}}\left(s,\tau \right)=10\log_{10}\left(P_{\mathrm{norm}}\left(s,\tau \right)\right)$$

### 2.7. Statistical Analysis

The primary goal of the statistical analysis was to characterize changes in the flexion–relaxation (FR) response after the prolonged cycling at both the individual and group levels.

The main outcome calculated was the FR ratio (FRR), computed for each lumbar muscle before and after the cycling protocol.

The FRR was computed using both time and time–frequency methodologies. In this study, time-domain sEMG envelopes were defined as the primary outcome measure. However, Wavelet-based frequency-domain features were analyzed as complementary exploratory descriptors to provide additional information on neuromuscular activation patterns, given its higher robustness to noise artifacts in the sEMG signals.

#### 2.7.1. Individual Analysis

Given that FR alterations are considered subject-specific neuromuscular phenomena [11], each participant was first classified according to the presence or absence of sEMG silence during maximal trunk flexion. Thus, participants were classified into

- Normal response (silent period present pre and post exercise).
- Persistent alteration (silent period absent in both pre and post exercise).
- Exercise induce alteration (silent period present in pre exercise but absent in post exercise).

The prevalence of altered responses was stimated as a binomial proportion with 95% confidence intervals calculated using the Clopper–Pearson method as seen in the following equations:

(10) $$p=\frac{x}{n},$$

where *x* represents the number of participants exhibiting altered FR responses and *n* the total sample size. Exact 95% confidence intervals were computed using the Clopper–Pearson method based on the cumulative binomial distribution:

(11) $$CI_{95\%}=\left[{\mathrm{Beta}}^{-1}\;\left(\frac{\alpha }{2};\;x,\;n-x+1\right),\;{\mathrm{Beta}}^{-1}\;\left(1-\frac{\alpha }{2};\;x+1,\;n-x\right)\right],$$

with α=0.05.

#### 2.7.2. Group Analysis

To complement the individual analysis mentioned previously, exploratory group comparisons were performed within the non-altered subjects to evaluate whether prolonged cycling induced neuromuscular changes. The purpose of this analysis was not to prove that cycling does change FRR but whether some individuals respond differently despite minimal average change.

For each muscle, pre to post exercise differences in FRR were summarized using the mean change:

(12) $$\Delta ={\bar{X}}_{post}-{\bar{X}}_{pre},$$

where ${\bar{X}}_{pre}$ and ${\bar{X}}_{post}$ denote the group mean values obtained before and after the cycling protocol, respectively.

The effect size was estimated using Cohen´s *d*, computed using the pooled standard deviation:

(13) $$d=\frac{\Delta }{SD_{\mathrm{pooled}}},$$

with the pooled standard deviation defined as

(14) $$SD_{\mathrm{pooled}}=\sqrt{\frac{SD_{pre}^{2}+SD_{post}^{2}}{2}}.$$

Approximate 95% confidence intervals (CI) for the mean difference were calculated as

(15) $$CI_{95\%}=\Delta \pm 1.96\;\frac{SD_{\mathrm{pooled}}}{\sqrt{n}},$$

where *n* represents the sample size of the non-altered subgroup.

#### 2.7.3. Statistical and Edition Software

All analyses were performed using MATLAB R2023a (MathWorks Inc., Natick, MA, USA).

An artificial intelligence-assisted language tool (OpenAI, GPT version 5.4) was used to improve the grammar and structure of the manuscript text.

## 3. Results

Three distinct neuromuscular response patterns were identified among the participants following the cycling protocol. First, the majority of subjects exhibited a clear myoelectric silent period both before and after exercise, indicating the absence of detectable sensorimotor disturbances. Second, a subgroup of participants did not demonstrate a myoelectric silent period in either assessment, suggesting a persistent absence of lumbar extensor inhibition throughout the protocol. This pattern was observed in three of the thirty-one volunteers. Third, one participant exhibited loss of the myoelectric silent period exclusively after exercise, indicating an exercise-related alteration of the flexion–relaxation (FR) response.

Importantly, the identification of altered FR patterns was not interpreted exclusively as exercise-induced changes, as some participants exhibited altered responses prior to the cycling protocol. Instead, these patterns were analyzed to characterize inter-individual variability in neuromuscular control under standardized loading conditions.

Overall, four of the thirty-one participants (12.9%) presented an altered FR pattern. The estimated prevalence of altered responses was 12.9% (95% CI: 3.6–29.8%), reflecting substantial inter-individual variability within the sample (Table 1).

**Figure 3:**
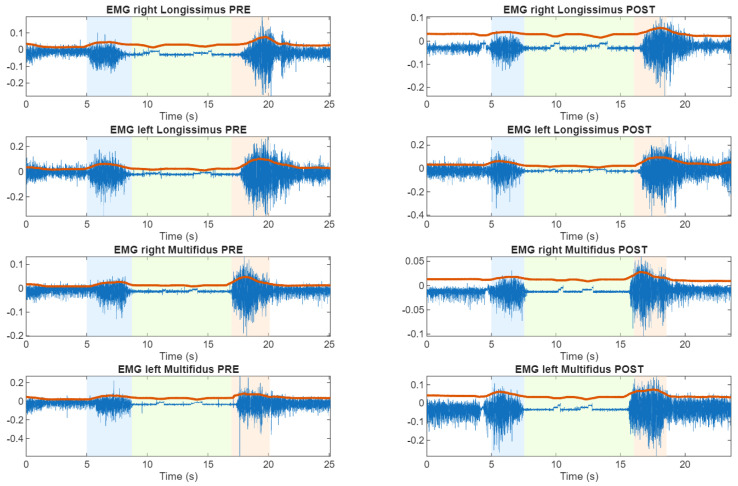
Time representations of lumbar sEMG activity during the flexion-relaxation test. Representative subject (S20) without sensorimotor disturbance showing a clear myoelectric silent period in both pre and post cycling exercise. Here, white area is the rest position, blue area is the flexion transition, green area is the maximum flexion, red area is the extension transition and red line is the envelope of the sEMG.

**Figure 4:**
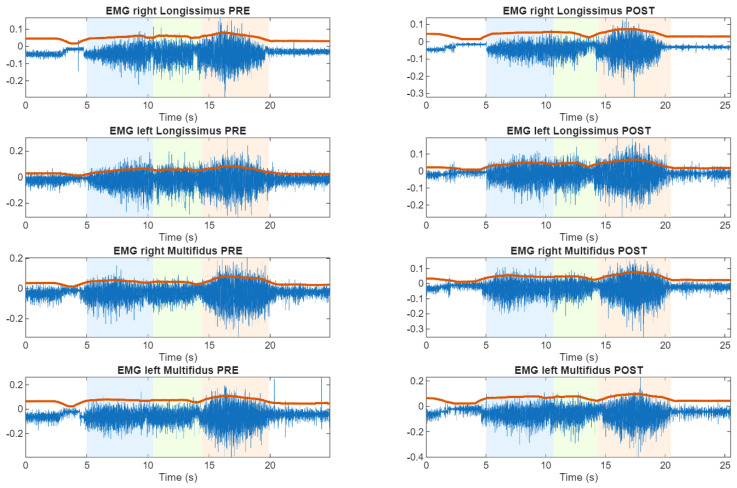
Time representations of lumbar sEMG activity during the flexion–relaxation test. Representative subject (S22) exhibiting persistent sensorimotor disturbance in both pre and post cycling exercise. Here, white area is the rest position, blue area is the flexion transition, green area is the maximum flexion, red area is the extension transition and red line is the envelope of the sEMG.

**Figure 5:**
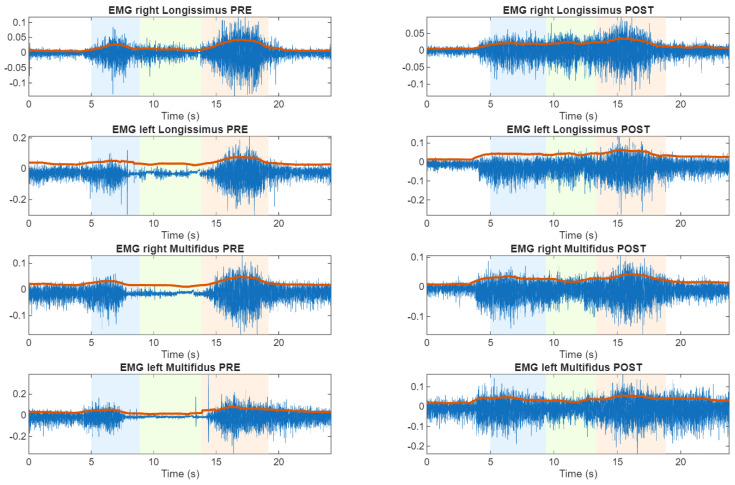
Time representations of lumbar sEMG activity during the flexion–relaxation test. Subject (S9) presenting loss of the silent period only after cycling, indicating an exercise-related alteration. Here, white area is the rest position, blue area is the flexion transition, green area is the maximum flexion, red area is the extension transition and red line is the envelope of the sEMG.

From the time analysis, and following the identification of the four participants that exhibit altered FR responses, we represent, as an example, the sEMG as shown in Figure 3, Figure 4 and Figure 5.

Descriptive summaries differentiating the average behavior of the non-altered group from the individual characterization of altered participants are presented in Table 2.

As summarized in Table 2, the RMS amplitudes and FRR values remained stable in the non-altered group.

**Figure 6:**
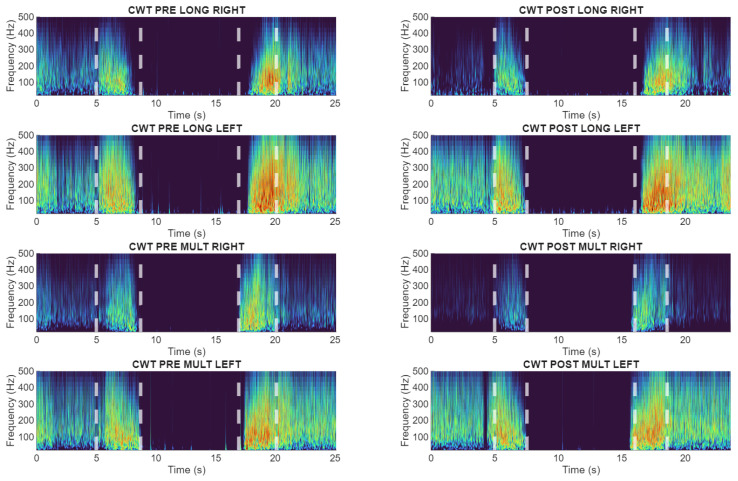
Wavelet-based time-frequency representations of lumbar sEMG activity during the flexion–relaxation test. Representative subject (S20) without sensorimotor disturbance showing a clear myoelectric silent period in both pre and post cycling exercise.

**Figure 7:**
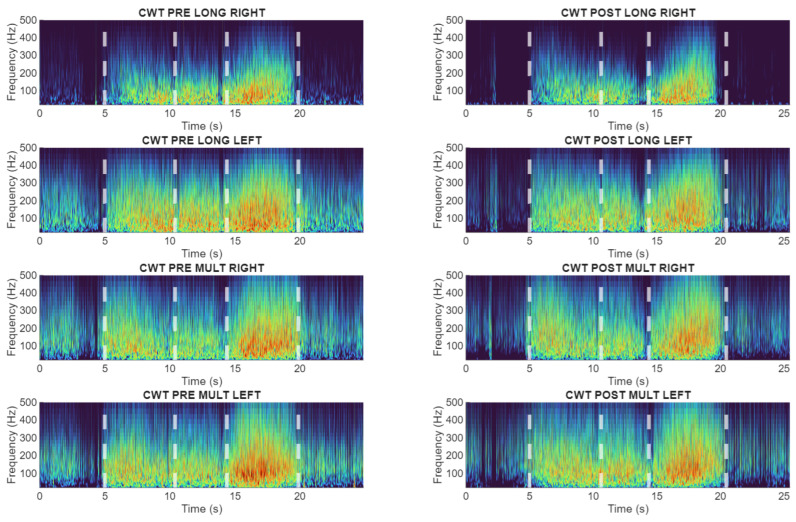
Wavelet-based time-frequency representations of lumbar sEMG activity during the flexion–relaxation test. Representative subject (S22) exhibiting persistent sensorimotor disturbance in both pre and post cycling exercise.

**Figure 8:**
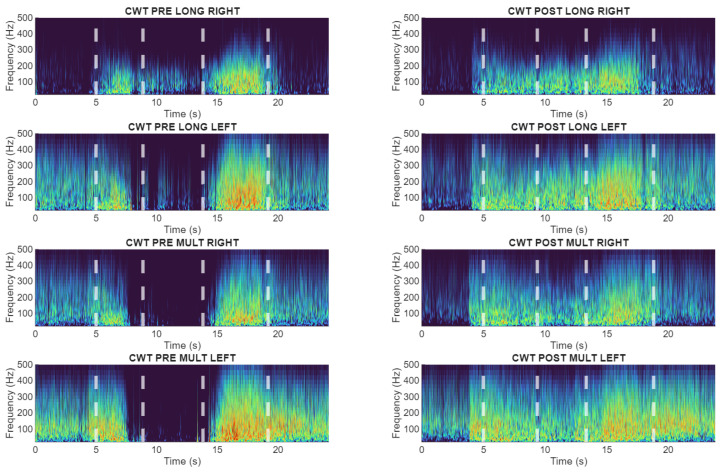
Wavelet-based time-frequency representations of lumbar sEMG activity during the flexion–relaxation test. Subject (S9) presenting loss of the silent period only after cycling, indicating an exercise-related alteration.

These response patterns were visually distinguishable in the wavelet frequency-domain representations (Figure 6, Figure 7 and Figure 8), which provided the clearest separation between preserved and altered neuromuscular behaviors.

Group-level statistical estimation within the non-altered subgroup (n=27) demonstrated minimal pre–post changes across all muscles. The mean differences (Δ) were computed as the difference between post and pre exercise FRR values, while the effect sizes were estimated using Cohen’s *d* based on the pooled standard deviation ($SD_{\mathrm{pooled}}$), and 95% confidence intervals (CI) were derived according to the equations described in Section 2.7.

For the right longissimus muscle, Δ=−0.19, and $SD_{\mathrm{pooled}}=0.97$, yielding d=−0.20 with 95% CI [−0.56,0.18]. For the left longissimus, Δ=−0.20, $SD_{\mathrm{pooled}}=1.27$, and d=−0.16 with 95% CI [−0.68,0.28]. The right multifidus showed a negligible increase (Δ=+0.03, $SD_{\mathrm{pooled}}=1.07$, d=0.03, 95% CI [−0.37,0.43]), while the left multifidus presented a similarly small change (Δ=+0.04, $SD_{\mathrm{pooled}}=1.07$, d=0.04, 95% CI [−0.36,0.44]).

Across muscles, the effect sizes were trivial, and the confidence intervals consistently crossed zero (Table 3), indicating the absence of a meaningful average modification of lumbar neuromuscular inhibition at the group level despite prolonged cycling exposure.

In contrast, participants with altered responses displayed heterogeneous but clearly abnormal patterns. Three individuals (S5, S15, and S22) showed FRR values close to unity in both evaluations, indicating sustained electromyographic activity during the relaxation phase and suggesting persistent impairment in lumbar extensor deactivation. Participant S9 exhibited a markedly different profile: high baseline FRR values declined substantially after cycling, reaching values at or below unity, consistent with loss of the flexion–relaxation phenomenon following exercise.

Across the altered subjects, the RMS and normalized sEMG amplitudes tended to exceed those observed in the non-altered reference group, reflecting increased tonic activation during the relaxation phase. This pattern was consistently observed across all muscle measurements.

Comparison between the flexion–relaxation ratio (FRR) and the extension–relaxation ratio (ERR) indicated that the FRR provided superior discrimination of neuromuscular behavior. Because extension inherently involves higher sEMG activation, the ERR demonstrated reduced sensitivity for detecting abnormalities during the relaxation phase and was therefore not used as the primary outcome.

**Figure 9:**
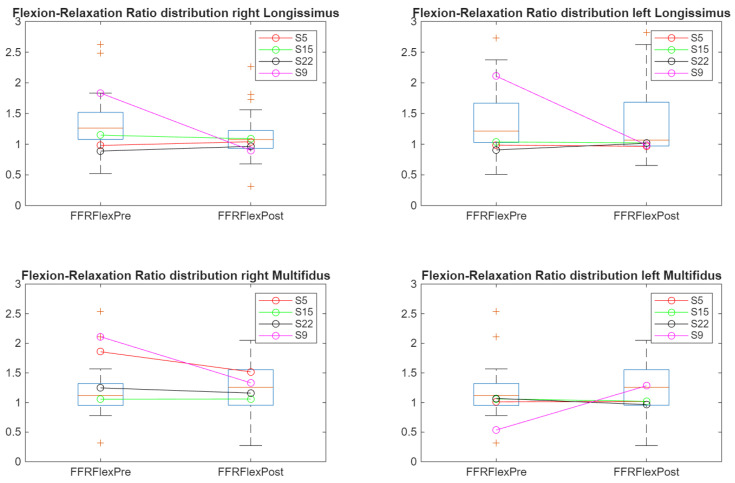
Time-domain flexion–relaxation ratio (FRR) trajectories before and after cycling across lumbar muscles. Individual altered participants are highlighted. “+” indicates outliers.

The FRR values were computed using both time-domain (mean sEMG envelope) and frequency-domain (wavelet-based RMS) features. Figure 9 illustrates time-domain FRR trajectories ranging approximately between 0.5 and 2. Participants lacking myoelectric silence in both assessments (S5, S15, and S22) displayed nearly horizontal trajectories around FRR ≈1, whereas S9 exhibited a pronounced downward shift across multiple muscles after cycling, indicating exercise-related alteration. An opposite trend observed in the left multifidus likely reflects residual signal artefacts affecting envelope estimation. This was due to motion-related noise, which, although notably reduced by the application of the Hampel filter (see Section 2.6.1), persisted over time in some cases. Nevertheless, most of the signals showed sufficient quality to be processed.

**Figure 10:**
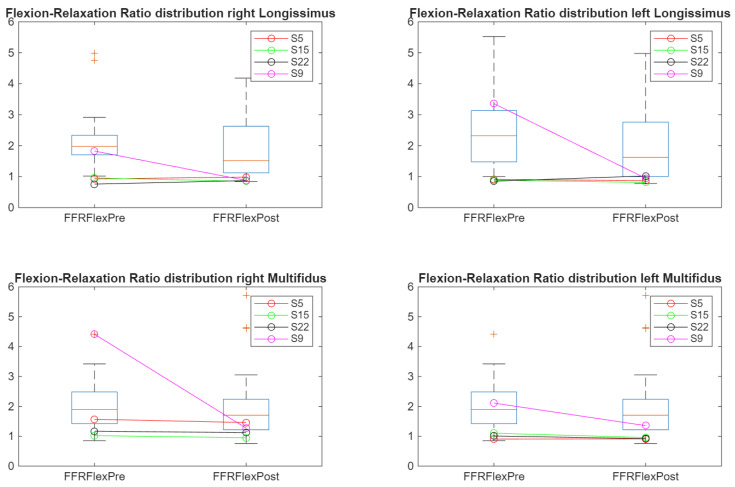
Wavelet-derived FRR distributions showing frequency-domain confirmation of neuromuscular behavior across pre and post exercise conditions. “+” indicates outliers.

Figure 10 shows patterns consistent with the time-domain analysis. Notably, the wavelet-derived FRR values revealed the expected negative slope in the left multifidus during post-exercise assessment, supporting the improved robustness of frequency-based estimation. Because the wavelet transform isolates spectral content independently of amplitude fluctuations, RMS values derived from wavelet coefficients provided a more stable indicator of neuromuscular inhibition across phases.

Visual inspection and quantitative analyses consistently enabled identification of the presence or absence of the myoelectric silent period during the relaxation phase. The absence of this silent period corresponded to altered neuromuscular behavior, whereas its preservation reflected normal lumbar extensor inhibition.

## 4. Discussion

This study suggests that a prolonged submaximal cycling protocol, performed under individualized and ecologically valid conditions based on VT1 and VT2, can reveal sensorimotor alterations in the flexion–relaxation (FR) response. Unlike previous protocols that incorporate static flexion or standardized cycling configurations, our design reproduces the sustained flexed posture and continuous power production characteristic of road cycling and integrates an objective measure of neuromuscular control before and after a physiologically consistent workload.

Four of the thirty-one participants (12.9%) presented an altered FR pattern. In three individuals, the alteration was present both before and after the protocol, while in one case it emerged only after exercise. Importantly, the altered FR pattern is characterized by a failure to suppress electromyographic activity at maximal flexion, indicating impaired inhibitory modulation in lumbar extensors. This finding is clinically relevant, because preservation of the FR silent period reflects the ability of the neuromuscular system to transition between passive and active stabilization modes. Its loss or reduction has been linked to altered sensorimotor control and reduced segmental stability.

A key aspect of the present findings is that most of the identified altered FR patterns were already present prior to the cycling protocol, with only one participant showing an exercise-associated change. This indicates that the primary contribution of this study is not to demonstrate a consistent exercise-induced alteration but rather to characterize the presence of subject-specific neuromuscular patterns under controlled physiological loading conditions.

Accordingly, the present results do not support the conclusion that prolonged cycling systematically alters lumbar neuromuscular control in pain-free cyclists. However, they do demonstrate that alterations in sensorimotor behavior, as assessed by the flexion–relaxation test, can be identified in a subset of asymptomatic individuals under these conditions.

Participants who exhibited altered responses both before and after the protocol likely reflect pre-existing neuromuscular patterns, potentially associated with accumulated exposure to cycling-specific mechanical demands over time [7,11,27].

In contrast, the observation of an exercise-associated change in one participant suggests that certain individuals may present increased sensitivity to sustained flexion under load, even within a relatively controlled and time-limited protocol. Considering that real-world cycling sessions frequently exceed the duration used in this study, it is plausible that longer or more demanding exposures could further accentuate these responses, as previously suggested in studies examining fatigue, creep, and load accumulation in the lumbar spine [19,20].

The small group-level changes observed should be interpreted cautiously, as averaging may obscure subject-specific neuromuscular responses, which constitute the primary focus of this study.

Therefore, while causal inference remains limited, the present findings contribute to the literature by providing evidence that the flexion–relaxation test, applied in a controlled yet ecologically informed context, is capable of detecting inter-individual differences in neuromuscular control in pain-free cyclists. This supports its potential utility as a complementary assessment tool to identify subject-specific neuromuscular behaviors that may not be captured through group-level analysis alone.

It is important to acknowledge that only four participants exhibited altered FR responses, which limits the strength of any generalizable conclusions. Although these cases provide relevant insight into individual neuromuscular behavior, the small number of altered observations and the associated uncertainty preclude strong claims regarding prevalence, early detection, or biomarker applicability. Accordingly, the findings should be interpreted as exploratory and descriptive, emphasizing the identification of subject-specific neuromuscular patterns rather than establishing definitive evidence of early detection or predictive clinical value.

The detection of altered FR responses in pain-free cyclists indicates that neuromuscular dysfunction and pain are not equivalent phenomena and may appear at different stages of exposure. This contrasts with the prevalence of LBP reported in cyclists, which ranges from 30% to 60% [5,6,7]. The lower prevalence observed here suggests that FR assessment identifies a different stage of the clinical continuum, in which sensorimotor alterations can emerge before pain perception.

Although the FR response has been proposed as a potential indicator of neuromuscular function, the present findings should be interpreted with caution regarding its role as an early biomarker. In the current study, altered patterns were identified in a subset of asymptomatic individuals; however, most of these patterns were already present prior to the cycling protocol, suggesting that they may reflect pre-existing neuromuscular strategies rather than acute exercise-induced changes. This is consistent with previous evidence indicating that alterations in motor control may exist independently of pain and may represent different stages within a broader sensorimotor continuum [10,11].

From this perspective, the flexion–relaxation test may be better understood as a tool capable of detecting individual neuromuscular behaviors under controlled conditions, rather than as a direct predictor of future low back pain. Its potential clinical relevance may lie in identifying subject-specific patterns that emerge in response to sustained loading conditions, which are characteristic of cycling practice and have been associated with cumulative mechanical stress and neuromuscular adaptations in the lumbar spine [7,19,20,27].

Although the cycling protocol may have induced peripheral fatigue, most participants did not exhibit alterations in flexion–relaxation behavior after exercise. This heterogeneous response suggests that post-exercise changes are not solely attributable to fatigue effects but may reflect subject-specific neuromuscular or sensorimotor characteristics. However, because no localized spectral fatigue analysis (e.g., median frequency shifts [31]) was performed, the contribution of peripheral fatigue cannot be completely ruled out.

Although participants were instructed to maintain a sustained flexed trunk posture and a fixed hand position during the cycling protocol, no continuous kinematic monitoring of lumbar spine angle was performed. Consequently, gradual postural adjustments or “postural creep” during the prolonged task cannot be excluded as potential contributors to the observed sEMG variations. Future studies should incorporate continuous motion tracking to objectively quantify the trunk posture and better distinguish fatigue-related or sensorimotor effects from biomechanical exposure differences.

The normalization of sEMG signals was performed using an MVC obtained in a static trunk-flexion posture, which may not fully represent muscle activation under dynamic gravity-assisted conditions. This approach could introduce limitations when interpreting absolute EMG amplitudes. However, the analyses performed in this study were based on relative intra-signal comparisons between flexion and relaxation phases, rather than on absolute amplitude values. Therefore, the normalization method is unlikely to have significantly influenced the identification of flexion–relaxation patterns.

Several biomechanical and exercise-related variables were not strictly controlled in the present protocol, including individual bicycle fit configuration, cadence regulation, and trunk movement strategies during cycling. These factors are known to influence lumbar muscle activation patterns and may therefore act as potential confounders when interpreting flexion–relaxation responses. The decision to allow self-selected cycling conditions was intended to enhance the ecological validity; however, future studies should consider more precise control or monitoring of these parameters, including standardized bike fitting procedures, cadence feedback systems, and quantitative assessment of trunk kinematics.

## 5. Clinical Implications

These findings provide clinically relevant information for the assessment and monitoring of neuromuscular behavior in cyclists exposed to sustained flexed postures. As none of the participants reported lumbar pain, the absence of symptoms does not necessarily reflect preserved neuromuscular control [32], and the identification of altered flexion–relaxation responses in a subset of asymptomatic individuals suggests the presence of subject-specific sensorimotor patterns under controlled loading conditions.

The FR test offers a non-invasive and objective measure of lumbar neuromuscular function. Rather than serving as a definitive biomarker of neuromuscular vulnerability, the present findings support its potential role as a complementary assessment tool to characterize individual neuromuscular strategies in cyclists [10,11].

From a practical perspective, the detection of altered FR patterns may contribute to a more comprehensive clinical evaluation when interpreted alongside biomechanical, training-related, and postural factors such as lumbo-pelvic stability, cycling posture, bike-fit configuration, and load exposure.

## 6. Conclusions

Prolonged cycling performed under physiologically controlled and ecologically valid conditions was associated with the emergence of subject-specific flexion–relaxation response alterations in the lumbar spine, even in the absence of pain. The alteration in the FR response, characterized by reduced or absent sEMG silence at maximal flexion, reflects variability in neuromuscular inhibition patterns and trunk motor strategies under sustained flexed postures rather than a definitive deficit in segmental stability.

These findings highlight the potential value of FR assessment as a complementary non-invasive tool to characterize individual neuromuscular responses to prolonged cycling exposure. However, given the limited number of altered cases and the exploratory nature of the analysis, further research with continuous kinematic monitoring and longitudinal designs is required to clarify its clinical and performance-related relevance in road cyclists.

## Figures and Tables

**Table 1:** Classification of flexion–relaxation (FR) responses before and after the cycling protocol.

Subject	PRE FR	POST FR	Type
S5	Absent	Absent	Persistent alteration
S9	Normal	Absent	Exercise-induced alteration
S15	Absent	Absent	Persistent alteration
S22	Absent	Absent	Persistent alteration
Non-altered group (n=27)	Normal	Normal	Normal neuromotor pattern

**Table 2:** Electromyographic variables during the relaxation phase of the flexion–relaxation (FR) test before and after cycling. Values are presented for the non-altered reference group (n=27) and participants exhibiting FR alterations (S5, S9, S15, S22).

Subject	Muscle	RMS Relax PRE	RMS Relax POST	Mean MVC Relax PRE	Mean MVC Relax POST	FRR RMS PRE	FRR RMS POST
Non-altered group (n=27)							
	R. Long	0.0046 ± 0.006	0.0049 ± 0.004	0.016 ± 0.016	0.016 ± 0.017	2.30 ± 1.34	1.84 ± 0.90
	L. Long	0.0047 ± 0.004	0.0045 ± 0.004	0.017 ± 0.016	0.016 ± 0.012	2.31 ± 0.96	2.17 ± 1.36
	R. Mult	0.0046 ± 0.006	0.0051 ± 0.007	0.016 ± 0.018	0.018 ± 0.016	2.08 ± 1.05	2.04 ± 1.28
	L. Mult	0.0051 ± 0.005	0.0057 ± 0.008	0.018 ± 0.017	0.019 ± 0.018	2.10 ± 1.05	2.06 ± 1.37
Participants with FR alterations							
S5	R. Long	0.095	0.079	0.096	0.100	0.90	1.00
	L. Long	0.064	0.066	0.066	0.062	0.66	0.96
	R. Mult	0.164	0.136	0.179	0.106	1.38	1.11
	L. Mult	0.236	0.229	0.178	0.172	1.08	1.07
S9	R. Long	0.620	0.621	0.003	0.004	1.81	0.69
	L. Long	0.629	0.593	0.003	0.004	3.38	0.96
	R. Mult	0.676	0.735	0.007	0.004	4.47	1.38
	L. Mult	0.709	0.945	0.006	0.006	2.89	1.29
S15	R. Long	0.145	0.110	0.009	0.015	1.34	1.04
	L. Long	0.197	0.191	0.096	0.078	1.83	1.20
	R. Mult	0.095	0.083	0.071	0.076	1.06	1.04
	L. Mult	0.095	0.099	0.097	0.099	1.00	1.07
S22	R. Long	0.094	0.064	0.009	0.008	0.98	0.90
	L. Long	0.084	0.060	0.007	0.008	0.98	0.96
	R. Mult	0.065	0.057	0.007	0.007	1.24	1.16
	L. Mult	0.083	0.089	0.006	0.008	1.06	0.96

**Table 3:** Pre–post changes in flexion–relaxation ratio (FRR) in the non-altered group (n=27).

Muscle	PRE Mean ± SD	POST Mean ± SD	Mean Difference	Cohen’s *d*	95% CI
Right Longissimus	2.13 ± 0.94	1.94 ± 0.99	−0.19	−0.20	[−0.56, 0.18]
Left Longissimus	2.37 ± 1.16	2.17 ± 1.36	−0.20	−0.16	[−0.68, 0.28]
Right Multifidus	1.97 ± 0.84	2.00 ± 1.26	0.03	0.03	[−0.37, 0.43]
Left Multifidus	2.01 ± 0.95	2.05 ± 1.17	0.04	0.04	[−0.36, 0.44]

## Data Availability

The datasets presented in this article are not readily available because the data are part of an ongoing study. Requests to access the datasets should be directed to David Arriagada-Tarifeño (david.arriagada@usach.cl).
